# A Meta-Analysis of the “Erasing Race” Effect in the United States and Some Theoretical Considerations

**DOI:** 10.3389/fpsyg.2020.01635

**Published:** 2020-08-26

**Authors:** Michael A. Woodley of Menie, Michael D. Heeney, Mateo Peñaherrera-Aguirre, Matthew A. Sarraf, Randy Banner, Heiner Rindermann

**Affiliations:** ^1^Center Leo Apostel for Interdisciplinary Studies, Vrije Universiteit Brussel, Brussels, Belgium; ^2^Independent Researcher, Charlotte, NC, United States; ^3^Department of Psychology, The University of Arizona, Tucson, AZ, United States; ^4^Independent Researcher, Boston, MA, United States; ^5^Department of Psychology, Technische Universität Chemnitz, Chemnitz, Germany

**Keywords:** alliance detection system, coalition, erasing race, social identity, meta-analysis

## Abstract

The “erasing race” effect is the reduction of the salience of “race” as an alliance cue when recalling coalition membership, once more accurate information about coalition structure is presented. We conducted a random-effects model meta-analysis of this effect using five United States studies (containing nine independent effect sizes). The effect was found (ρ = 0.137, *K* = 9, 95% CI = 0.085 to 0.188). However, no decline effect or moderation effects were found (a “decline effect” in this context would be a decrease in the effect size over time). Furthermore, we found little evidence of publication bias. Synthetically correcting the effect size for bias stemming from the use of an older method for calculating error base rates reduced the magnitude of the effect, but the it remained significant. Taken together, these findings indicate that the “erasing race” effect generalizes quite well across experimental contexts and would, therefore, appear to be quite robust. We reinterpret the theoretical basis for these effects in line with Brunswikian evolutionary-developmental theory and present a series of predictions to guide future research in this area.

## Introduction

Historically, various conceptualizations of “race”^1^ as corresponding to or capturing population structure or taxonomic categories among a group of individuals who share ancestry have been proposed; these are often used as synonyms for “subspecies” when applied to human taxonomy (e.g., Garn, 1961). The application of such concepts to humans has nevertheless proven highly controversial (Keita, 1993, cf. Sesardić, 2010). More recently, a body of sociological theory has challenged historical biologized conceptions of “race” and has advanced the idea that “race” is a purely socially constructed phenomenon, functioning as a source of personal identity and (in some cases) also social privilege (Zack, 2018). Such arguments have also tended to draw on the observation that there is more genetic variation within “races” than between them, which has been employed as evidence that “races,” as historically conceptualized in anthropology, are taxonomically meaningless and that the concept persists for social and cultural reasons [Lewontin, 1972; but see the criticisms of Lewontin’s argument from Reich (2018)].

Population geneticists tend now to use the less loaded terms “continental population” or “biogeographic ancestry group” to refer to the high-level genetic population structure that arises from cluster analyses of different gene frequencies within the human species (e.g., Cavalli-Sforza, 2000). Matters surrounding the use of the term “race” remain controversial in both scientific and, more broadly, public discourse concerning the nature of personal identity, social power, and ancestry (Zack, 2018).

Indeed, the broader scientific debate about the role of social versus biogeographical factors in the construction of “race” has inspired interesting lines of research, such as that which has focused on the degree to which “race” functions as an innate coalitionary alliance cue. In line with the greater historical prevalence of biological thinking on the nature of “race” (Littlefield et al., 1982), it had been assumed that race (along with age and sex) is a high-salience coalition cue that is automatically encoded for the purposes of alliance detection (Taylor et al., 1978; Fiske and Neuberg, 1990; Stangor et al., 1992; Hamilton et al., 1994). Kurzban et al. (2001) offered a substantial challenge to this assumption. In the light of advances in the field of evolutionary psychology, understanding of the evolved bases of human behavior changed dramatically, and certain novel theoretical insights gave reason to doubt older views about the salience of “race” to alliance detection.

The model advanced by Kurzban et al. (2001) posits that selection pressures present (mostly) in the Pleistocene Epoch shaped the adaptively salient facets of the human mind [the Pleistocene corresponds to the period approximately 2.58 million to 11,700 years before the present (ybp), and “modern” humans originated approximately 300,000 to 200,000 ybp]. The spatially and temporally contiguous set of environments that shaped the adaptive architecture of modern humans is collectively referred to as the *environment of evolutionary adaptedness* or EEA (Barkow et al., 1992). It has been argued that in the EEA, selection pressures were recurrent and involved domain-specific fitness challenges. These challenges tended to favor the evolution of *specialized* psychological adaptations, or modules, which are dedicated, evolved psychological mechanisms for dealing with specific problems. Evolved modules are thought to underpin psychological and behavioral phenomena, such as kin recognition, discriminative parental solicitude, disgust sensitivity, incest avoidance, language acquisition, and cheater detection, among others (Buss, 2011).

Human populations living in the (majority of the) EEA had a (primarily) hunter–gatherer subsistence paradigm. The main out-of-Africa event for *Homo sapiens* is thought to have started around 70,000 ybp. It is assumed that, owing to the presence of a breeding structure characterized by limited spatial dispersal, contact between individuals of the subsequent spatially isolated populations, especially during the Pleistocene, was extremely infrequent, preventing the evolution of a dedicated “racial” coalition module (insofar as the resultant populations could be said to correspond to folk or even anthropological notions of “race”). Kurzban et al. (2001) went further, intimating that the concept of “race” is inapplicable to non-polytypic taxa such as *Homo sapiens*, in which there is far more genetic variation within than between populations (Lewontin, 1972), coupled with an “at most geographically graded” (p. 15387), as opposed to “sharply bounded” (p. 15387) distribution of the latter. Therefore, assuming (1) infrequent encounters between members of geographically separate populations in the EEA and (2) minimal correspondence between “races” and actual genetic population structuring, “racial” and ethnic cues to coalition formation likely are not evolutionarily encoded in humans’ perceptions of coalition membership. The use of “race” for the purposes of coalitional categorization might therefore be a by-product of the way in which certain modules use arbitrary, but stable, appearance-related cues as a basis for alliance detection. Further, given their arbitrary nature, the influence of prospective badges of “race” and ethnicity should be especially weak when other more socially salient cues are present.

To test this model, Kurzban et al. (2001) conducted a variant of the “who-said-what” type of recall study (Taylor et al., 1978) involving 107 student participants who were given images of individuals broken out by “race” (Black and White) and were asked to assign them to one of two basketball teams after being given a limited amount of time to read a sequence of antagonistic statements associated with each individual, which were presented as part of an argument between two rival teams. In one condition (visual cue absent), the individuals in the photographs all wore t-shirts of one color (either gray or yellow), and in the second condition (visual cue present) the students wore t-shirts of different colors, corresponding perfectly to their team membership (gray and yellow). After exposure to a distractor (listing as many United States as possible), the participants were then asked to recall team membership for each individual by matching a sentence to the individual. Attribution errors were coded for both conditions. It was found that students tended to utilize “race” (i.e., Black or White) as a coalition marker under the visual cue absent condition more often than under the visual cue present condition—in which they tended to make accurate attributions based on t-shirt color instead—this despite the fact that the sentences contained sufficient information to assign individuals to teams in both conditions. In other words, the salience of “race” to coalition was reduced (or “erased” to use Kurzban et al.’s term) in the presence of an alternative and more accurate visual cue. One would not expect these results if the salience of “race” to coalition formation was evolutionarily encoded in human psychology. Further experiments provided support for this expectation. Specifically, variants of the experiment that involved mixed-sex targets revealed that sex was consistently more salient (i.e., tended to be coded more frequently upon recall) to coalition assignation across conditions. These findings on the whole align with the prediction that sex is an evolutionarily highly salient phenomenon but that “race” is not.

The theoretical underpinnings of the Kurzban et al. (2001) model have been critiqued, most recently by Salter and Harpending (2013), who have proposed that cooperation among co-ethnics has the potential to yield very substantial fitness payoffs that scale in proportion to the level of genetic differentiation between competing groups. Furthermore, they argue that erroneous evolutionary assumptions limit the generalizability of Kurzban et al.’s theory. The first problematic assumption is Kurzban et al.’s (2001) reliance on Lewontin’s argument that “the overwhelming preponderance of genetic variation is within population and not between population” (p. 15387) as a basis for diminishing the applicability of the “race” concept to the apportioning of taxonomic diversity within *H. sapiens*. This is countered with reference to work finding that in considering correlations across multiple genetic loci [rather than conducting a “locus-by-locus” analysis as Lewontin did (Edwards, 2003, p. 799)], patterns of variance apportionment indicative of taxonomically meaningful structuring in the human species emerges (Dawkins, 2004, pp. 406–408, Edwards, 2003; Tal, 2012). When examined in relation to autosomal markers of biogeographic ancestry, such correlation structures can be cladistically meaningful, and even allow highly accurate prediction of individuals’ self-identified “race” (Tang et al., 2005; Guo et al., 2014). The second problematic assumption is that ancestral human populations living in the EEA were too spatially isolated to have come into regular contact with one another. Salter and Harpending (2013) suggest instead that there is a “high likelihood that regular contact of very different peoples occurred over most of human history, with “fully modern humans” being “only 45,000 years old” (p. 259). This model is based on the idea that, for most of their evolutionary history, discrete human populations would radiate outward from their point of origin, repeatedly coming into contact and potentially also conflict with one another (see Harpending and Harris, 2016).

This model, coupled with the observation that fitness payoffs to cooperation among co-ethnics scale in proportion to the degree of genetic differentiation between competing groups, implies that contact among distinct biogeographic ancestry groups may have been both frequent and costly enough (in instances when it involved conflict) to constitute an adaptive problem over much of the history of modern humans.

Theoretical criticisms such as these, along with the claim that Kurzban et al.’s (2001) original finding may lack generalizability (Salter and Harpending, 2013), invites meta-analytic scrutiny, whereby the robustness of the “erasing race” effect can be assessed across studies and across experimental contexts. Such scrutiny of the “erasing race” effect is also warranted given the replication crisis in social and experimental psychology (Pashler and Wagenmakers, 2012). Findings supporting certain foundational claims in evolutionary psychology, such as the ovulatory shift hypothesis, have also failed to replicate recently (e.g., Jones et al., 2018a, b); thus, this field may well not be immune to the crisis. In light of these considerations, we utilize formal meta-analysis to examine the robustness of effects involving studies that broadly replicate the original Kurzban et al. (2001) approach—specifically all of those studies in which strong visual cues to coalition membership were crossed with participant “race” in order to observe the change in “race”-based categorization errors.

## Materials and Methods

A meta-analysis was conducted following the Preferred Reporting Items for Systematic Reviews and Meta-Analyses (PRISMA) guidelines (Moher et al., 2009). To that end, full details of study selection–exclusion criteria are reported along with a flowchart illustrating how the original study pool was reduced to the final set of effect sizes. All references to selected studies are indicated with asterisks in the reference section. Finally, all data utilized in this analysis are also reported.

### Search Strategy

The literature search began through the PsycINFO, PsycArticles, and Academic Search Complete databases, performed simultaneously through the utilization of EBSCOhost. Based on an inspection of the relevant known articles, the following keywords were used to obtain our initial study pool: “social categorization,” “coalitional psychology,” “racial encoding,” “who said what?” or “memory confusion protocol,” “erasing race” in conjunction with “race” or “ethnicity.” ProQuest Dissertations and Theses Global was searched separately utilizing the same keywords. After the initial pool of articles was identified, manual reviews of the reference sections and forward and backward searches using the Social Science Citation Index were performed to identify any additional studies not found in the previous database searches. Prominent experts in the field were contacted via email to identify any unpublished data or any studies currently in press. As an extra measure, we searched the vitae of prominent authors to confirm that no unpublished results were available. Two unpublished presentations that likely qualified for the current study were identified in this manner, one based on Brazilian participants was requested but not provided by the authors (Cosmides et al., 2012), who stated that the reported effects needed to first be recalculated according to an improved error base rate correction methodology identified by Bor (2018) and discussed by Pietraszewski (2018); the implications of this for the present effort are discussed in detail in subsequent sections. A second analyzed a small sample of UCLA students, investigating the “erasing race” effect for males and females separately in addition to examining the effects of primes and individual differences correlates of performance (Moya et al., 2005). The lead author was contacted and made all relevant data available to us. This set of effects could, therefore, be incorporated into the present meta-analysis. Thus, our meta-analysis is restricted to only studies conducted with United States participants.

### Inclusion/Exclusion Criteria

This review included studies in the English language that were published between January 2014 and January 2019. A search prior to 2014 was not required because one study (Voorspoels et al., 2014) surveyed all (known to those researchers) articles prior to 2014 for which data were available for reanalysis. The only pre-2014 study that had been missed in Voorspoels et al.’s own search of the literature (Moya et al., 2005) had already been found via contact with topic experts. Studies needed to report quantitative results from “who said what?” experiments that crossed coalition with “race” under visual cue versus no visual cue conditions, comparable to the results produced in Kurzban et al. (2001) as described above. Recognizing *a priori* that the volume of comparable empirical literature is very small (and direct replications of the Kurzban et al. study smaller still), we thoroughly reviewed any article that conforms to these relatively broad conditions and established methodological moderators to account for any deviations in experimental designs.

**FIGURE 1:**
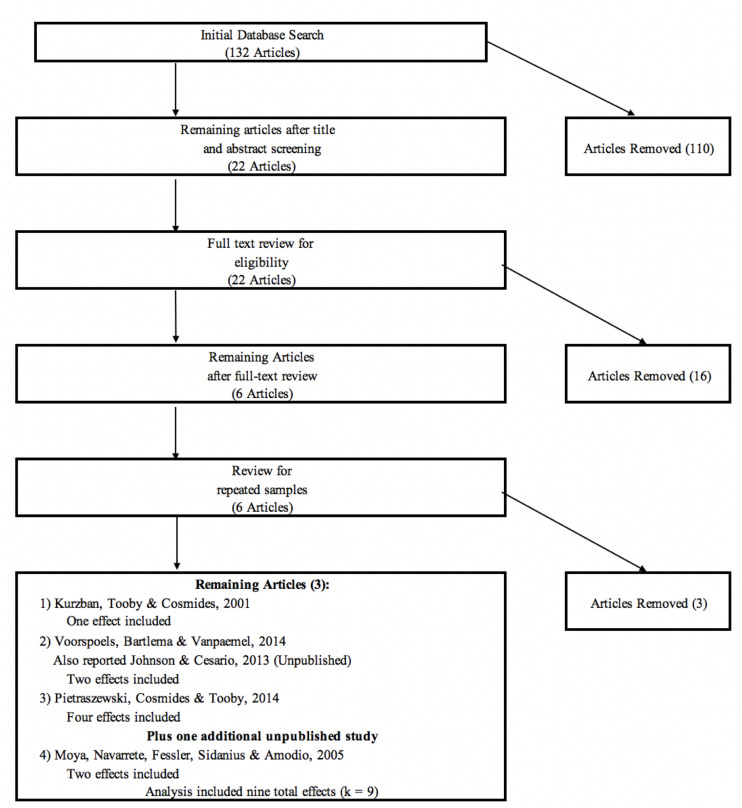
PRISMA flowchart illustrating the literature selection procedure. The original study pool included 132 articles; after filtration based on the inclusion rules, the final study pool contained five studies and a total of nine distinct effect sizes.

Upon collecting the initial pool of articles, studies were screened that met the above criteria and reported effect sizes in the form of bivariate correlations, *t* statistics, Cohen’s *d*, or other effect-size statistics that can be converted to bivariate correlations. As depicted in Figure 1, we began with 132 articles from the initial electronic search. After reviewing the abstracts, 110 articles that were clearly unrelated to our general topic were eliminated. Examples of excluded articles include studies of multiracial targets, facial expressions of emotion, attractiveness, and neurological studies that were far removed from Kurzban et al.’s experimental conditions. Twenty-two articles indicated some potential for inclusion and were retained for a detailed narrative review. Following these reviews, an additional 16 articles were eliminated. Of the six remaining articles (including Kurzban et al.), it became apparent upon further review that four articles reported statistics from a common sample (reported originally in Pietraszewski, 2009), necessitating the exclusion of three articles to avoid issues with repeated measures. Of the four articles, Pietraszewski et al. (2014) reported the most detail from which comparable effects could be calculated, which supports our decision to retain this article and exclude the others. The result of our search ultimately yielded nine distinct effect sizes. Effects from three studies (including Kurzban et al.) were reported in Voorspoels et al. (2014), two compatible results from one unpublished study (Moya et al., 2005) were made available to us on request, and the remaining four effects were calculated from the results reported in Pietraszewski et al. (2014).

### Moderators

#### Antagonistic vs. Non-antagonistic

Experimental conditions in Pietraszewski et al. (2014), although meeting our criteria for selection, differ from the Kurzban et al. framework in two primary ways that can be easily accounted for thorough moderator analysis. Most notably, Kurzban et al. (2001), along with the two additional studies reported in Moya et al. (2005) and Voorspoels et al. (2014) presented participants with a scenario in which two competing basketball teams recently engaged in a fight during a game, and Pietraszewski instead presented two non-competing charities with the common goal of helping others. To address this difference, a moderator variable to indicate the presence or absence of *antagonism* in the presented scenarios was established. The three effects in Voorspoels et al. (2014) were coded “Yes” for antagonism, and the four effects from Pietraszewski et al. (2014) were coded “No.” In addition to reflecting the presence or absence of antagonism in the scenarios, this moderator also captures differences in coalition type (basketball teams vs. charitable groups) and differences in the content of the statements themselves. As a result, differences in the effect sizes between the two groups distinguished in the *antagonism* moderator should be interpreted according to the presence or absence of antagonism in conjunction with these other distinguishing features.

#### Experimental Condition

For the next moderator set, we calculated effects according to three experimental comparisons. The first comparison reflected the color shirt/no color condition under a verbal coalition cue present condition, this comparison most closely approximating the verbal cue conditions (i.e., in which clues to coalition membership were present in the statements given to participants to read) originally established by Kurzban et al. (2001), and employed by Johnson and Cesario (2013), Voorspoels et al. (2014), and Moya et al. (2005). At retrieval, the Kurzban condition statements retain some remnants of verbal cues (because the statements are identical to those presented in the encoding phase) although the statements were randomized in order to make it more difficult to infer coalition based on verbal cues alone; thus, despite the presence of verbal cues to coalition, an attempt was made to make coalition irrelevant at recall.

In a subset of experiments, Pietraszewski et al. (2014), however, utilized statements that are completely coalition neutral, being devoid of verbal cues altogether. This “no coalition” condition reflected different targets, male and female, Black and White, making innocuous statements that, although possible to associate correctly during the recall phase, were not particularly memorable in their content and provided nothing to indicate coalition. This condition was presented to distinct participants according to “shirt color” and “no color” (gray) conditions as described, thus establishing a useful “neutral” baseline against which other experimental conditions can be compared.

Pietraszewski et al. (2014) also generated two other conditions in which verbal coalition cues were present, but made irrelevant and were also made relevent. To elaborate, identical statements at encoding are provided under both conditions, in which a portion of each statement provides no indication of coalition membership, and another portion clearly indicates membership in one of two charities, Habitat for Humanity (through direct references to its mission of building homes) or Partners in Health (through direct references to its mission of eradicating hunger). Although the full statements are presented for both the coalition relevant and coalition irrelevant conditions at encoding, at recall portions of the same statements containing references to coalition were removed entirely under the coalition irrelevant condition, and only portions of the statements that directly reflect each coalitions’ mission were retained in the coalition relevant one.

Detailed results obtained from the Pietraszewski et al. (2014) supplement provided us with the ability to calculate four effect sizes corresponding to a coalition neutral (gray shirt) vs. coalition relevant (color shirt) comparison and a coalition irrelevant (gray shirt) vs. coalition irrelevant (color shirt) comparison, for both male and female targets separately. These particular permutations were chosen in order to heighten the contrast between experimental conditions. Additional permutations (i.e. coalition irrelevant vs. coalition relevant) were not coded so as to avoid issues related to repeated measures. Thus, for this moderator, we group effect sizes based on whether they employed the “Kurzban procedure” (i.e., color shirt/no color shirt, coalition relevant) (five effect sizes) or whether they combined the coalition neutral no color condition with the coalition relevant color condition, or whether they combined the coalition irrelevant no color with the coalition irrelevant color condition (two effect sizes per moderator).

#### Additional Moderators

Additional moderators were established as follows: (1) *student*, to distinguish whether the sample reflected students versus a more general population, (2) *published*, to distinguish whether the study was published or not, and (3) *participant sex*, to distinguish the separate male and female participant results reported in Moya et al. (2005) from the results of the other studies, which all utilized mixed-sex participants, and (4) *preregistered*, to distinguish the Voorspoels et al. (2014) study, which performed a power analysis to identify requisite sample sizes and that formally stated expected results *a priori*. Table 1 lists each study used along with its relevant characteristics.

**TABLE 1:** Study-level effects for five distinct “erasing race” studies, along with moderators.

Author(s)/year	Context	Antagonistic	Experimental condition	Participant sex	Preregistered	Published	Student	Target sex	Weighted *r*
Johnson and Cesario, 2013	Basketball Teams	Yes	Kurzban procedure	Both	No	No	Yes	Male	0.08
Moya et al., 2005	Basketball Teams	Yes	Kurzban procedure	Male and Female	No	No	Yes	Male	0.15
Kurzban et al., 2001	Basketball Teams	Yes	Kurzban procedure	Both	No	Yes	Yes	Male	0.18
Pietraszewski et al., 2014 (data collected c. 2009)	Charity Groups	No	Coalition neutral X coalition relevant and coalition irrelevant X coalition irrelevant	Both	No	Yes	Yes	Male and female	0.18
Voorspoels et al., 2014	Basketball Teams	Yes	Kurzban procedure	Both	Yes	Yes	No	Male	0.09

### Quantitative Analyses

The calculation of inputs to our meta-analysis began with the (reported) difference scores (means, standard deviations, and *N*s) between the same-“race” and different-“race” errors for the conditions that we targeted for comparison. The means, standard deviations, and *N*s of the reported difference scores were then input into the online Practical Meta-Analysis Effect Size Calculator referenced by Lipsey and Wilson (2001). This tool calculated the Pearson correlation coefficients that indicate the magnitude of the “erasing race” effect consistent with the effects reported in Voorspoels et al. (2014). Pearson correlation coefficients have the advantage of being good “intuitive” effect sizes, making the practical significance of effect sizes more apparent.

Study-level effects, sample sizes, and moderators were double-coded independently by two authors (MH and RB) using Excel spreadsheets and were then independently audited to ensure consistency in the coding between the two spreadsheets and to confirm the absence of any coding errors. All analyses were conducted using Comprehensive Meta-Analysis (CMA) Version 3 (Borenstein et al., 2015). The initial analysis estimated the weighted mean effect size and its distribution across studies. Within-group comparisons at the study level are reflected in a statistic of within-group variation, Q_*w*_. Between-group variation is indexed with Q_*b*_. Both Q-statistics follow a chi-square distribution similar to those applied in the analysis of individual samples. Consistent with established convention, the present study adopted an alpha level of 0.05. Results reported in this study are from the random effects model. A random effects model is appropriate when different experimental conditions and/or sample characteristics are expected to exist across the included studies, which is our assumption.

In addition to performing calculations of a weighted effect and moderator analyses according to the described moderator groups, we also performed a meta-regression that specified effect sizes as a function of study or data collection date to identify the presence or absence of any temporal trend. Dates assigned to each record correspond to the publication year (or year presented in the case of Moya et al., 2005; Johnson and Cesario, 2013) except for the effects reported from Pietraszewski et al. (2014) given that these data were first presented in 2009 as part of a doctoral dissertation (Pietraszewski, 2009).

Several procedures were performed to infer the presence or absence of publication bias. Publication bias can result when studies yielding null findings fail to be reported. To the extent that this occurs, conventional literature searches may overlook these studies causing a potential bias in the distribution of effect sizes examined (Borenstein et al., 2015). Although there is no way to account for publication bias directly, there are techniques to estimate the potential for this to occur based on the studies that were identified through the literature search. One method is through the use of a funnel plot. Publication bias is evident when the plot depicts an asymmetrical distribution of effects about the overall point estimate (represented by a vertical line), suggesting that studies of smaller sample sizes (and, therefore, greater standard error) and with large effects are preferred by publishers due to favorable outcomes (Borenstein et al., 2015). In addition, an Egger’s regression was performed to indicate whether the distribution of effects is symmetrical, which, if supported, suggests that any unidentified study results likely do not deviate significantly from our overall findings. We also performed a “trim and fill” analysis (Duval and Tweedie, 2000) to infer the presence of publication bias by estimating the number of unidentified effects required to achieve a completely symmetrical distribution of effect sizes.

Finally, as a robustness test, we attempted to synthetically correct the meta-analytic value for bias stemming from a recent methodological improvement in the error base rate calculation (Bor, 2018) using an aggregate estimate of the difference in 55 effect sizes (*r* values) computed using the old and the new method from data presented in the Pietraszewski (2018) online supplement.

## Results

### Main Analysis

The effect sizes associated with each study, along with a forest plot are presented in Table 2. All effects are positive in sign, but seven of them do not reach conventional significance (the lower 95% confidence interval [CI] bisects the zero line) when this effect size is estimated using study degrees of freedom. The possible loss in significance might stem from interconversion of effects between *d* and *r* (such conversions are known to very slightly bias values; Schmidt and Hunter, 2015) and from rounding down to two decimal places (so as to homogenize the reporting of each effect across studies), both of which may make marginally significant *d* values reported in one study marginally non-significant when recomputed for meta-analysis.

**TABLE 2:** Study-level effects, *N*, and confidence intervals for nine distinct “erasing race” effects, along with forest plot.

Author	Effect Size (*r*)	*N*	Lower C.I. (95%)	Upper C.I. (95%)	Forest Plot (Correlations and 95% C.I.)
Kurzban et al., 2001	0.180	107	–0.010	0.358	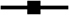
Voorspoels et al., 2014	0.090	463	–0.001	0.180	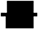
Moya et al., 2005 (M)	0.200	46	–0.096	0.463	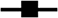
Moya et al., 2005 (F)	0.110	57	–0.155	0.360	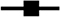
Johnson and Cesario, 2013	0.080	175	–0.069	0.226	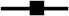
Pietraszewski et al., 2014 (1)	0.260	144	0.101	0.406	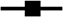
Pietraszewski et al., 2014 (2)	0.180	129	0.007	0.342	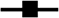
Pietraszewski et al., 2014 (3)	0.150	146	–0.013	0.305	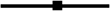
Pietraszewski et al., 2014 (4)	0.140	165	–0.013	0.287	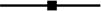
**Overall effect (random effects)**	**0.137**	**1432**	**0.085**	**0.188**	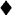
					**−1.00** **−0.50** **0.00** **0.50** **1.00** **Favors A** **Favors B**

The results of the main meta-analysis are presented in Table 3. The point estimates of the population correlation (ρ) indicate significant but small-magnitude effects (i.e., the effect size falls between 0.10 and 0.29; Cohen, 1988) when this is estimated using a random effects model. The *I*^2^ parameter indicates that 0.000% of the between-study variance is due to heterogeneity. The *Q* statistic also indicates non-significant heterogeneity, indicating that the studies are highly congruent with one another.

**TABLE 3:** Random effects models for the “erasing race” effect.

Model	Number Studies	Point estimate (ρ)	Lower limit (95% C.I.)	Upper limit (95% C.I.)	*z*-value	*Q*	*I*^2^
Random	9	0.137***	0.085	0.188	5.170	4.631	0.000

***p < 0.001.

### Moderation Analyses

The moderators were decomposed into substantive (i.e., those differences among studies that may stem from differences in sample characteristics, such as the use of student vs. population-level sampling) and methodological (i.e., those differences among studies that may stem from methodological differences, such as whether the study was preregistered or not). These are presented in Tables 4, 5.

**TABLE 4:** Point estimates and heterogeneity analyses for substantive moderators.

Analysis	*K*	*P*	Lower C.I. (95%)	Upper C.I. (95%)	*Q*_*b*_	*Q*_*w*_	*I*^2^
**Antagonistic**	9				1.99		
No	4	0.181***	0.101	0.259		1.41	0.000
Yes	5	0.106**	0.039	0.173		1.23	0.000
**Participant sex**	7				0.223		
Female	1	0.110	–0.155	0.360		4.41	0.000
Male	1	0.200	–0.096	0.463		0.00	0.000
Both	5	0.136***	0.083	0.189		0.00	0.000
**Student versus population**	9				1.56		
No	1	0.090	–0.001	0.180		0.000	0.000
Yes	8	0.160***	0.097	0.221		3.08	0.000

***p < 0.001, **p < 0.01.

**TABLE 5:** Point estimates and heterogeneity analyses for methodological moderators.

Analysis	*K*	*ρ*	Lower C.I. (95%)	Upper C.I. (95%)	*Q*_*b*_	*Q*_*w*_	*I*^2^
**Target sex**	9				0.159		
Female	2	0.158**	0.043	0.268		0.119	0.000
Male	7	0.137***	0.074	0.189		4.352	0.000
**Experimental conditions**	9				2.919		
Kurzban procedure	5	0.106**	0.039	0.173		1.232	0.000
Coalition Irrel. vs. Coalition Irrel.	2	0.145*	0.033	0.252		0.008	0.000
Coalition Neut. vs. Coalition Rel.	2	0.223***	0.106	0.333		0.471	0.000
**Published**	9				0.343		
No	3	0.105	–0.014	0.222		0.518	0.000
Yes	6	0.145***	0.087	0.201		3.770	0.000
**Pre-registered**	9				1.556		
No	8	0.160***	0.097	0.221		3.075	0.000
Yes	1	0.090	–0.001	0.180		0.000	0.000

***p < 0.001, **p < 0.01, *p < 0.05.

### Publication Bias Checks

**FIGURE 2:**
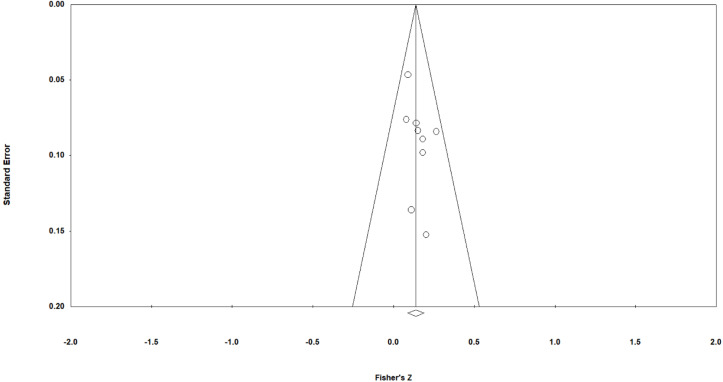
Funnel plot of standard error and Fisher’s *z* corresponding to study-level “erasing race” effect sizes. Each study is represented as a circle and the aggregate effect as a rhomboid. The diagonals are the 95% confidence interval control lines.

A funnel asymmetry plot was generated to examine the effects for indications of outlying values—this is graphed in Figure 2. The effects are symmetrically distributed around the midline with no effects falling outside of the 95% CI control lines.

The symmetry of the distribution is confirmed via the computation of the Egger’s regression (non-significant result indicates non-significant deviation from symmetry), presented in Table 6.

**TABLE 6:** Results of the Duval and Tweedie’s trim and fill test and the Egger’s regression analysis of symmetry.

Duval and Tweedie’s trim and fill								

		Fixed Effects			Random effects			
	Studies trimmed	Point estimate	Lower limit (95% C.I.)	Upper limit (95% C.I.)	Point estimate	Lower limit (95% C.I.)	Upper limit (95% C.I.)	*Q*
Observe*d* values		0.137	0.085	0.188	0.137	0.085	0.188	4.631
Adjusted values	4	0.106	0.061	0.151	0.107	0.061	0.151	11.290

**Egger’s regression intercept**								

**Intercept**	**Standard error**		**Lower limit (95% C.I.)**	**Upper limit (95% C.I.)**	***t*-value**	**Df**		***p*-value**

1.181	0.713		−0.505	2.867	1.656	7		0.145

Also presented in Table 6 are the results of the Duval and Tweedie (2000) trim-and-fill test, which determines the presence of publication bias by estimating the number of “missing” effect sizes that would be needed to achieve true symmetry in the distribution of the effect sizes. It was found that there were four “missing” effect sizes to the left of the mean, suggesting that, had they been accounted for, the overall effect would have reduced to 0.106, which would still have yielded a statistically significant meta-analytic aggregate.

**FIGURE 3:**
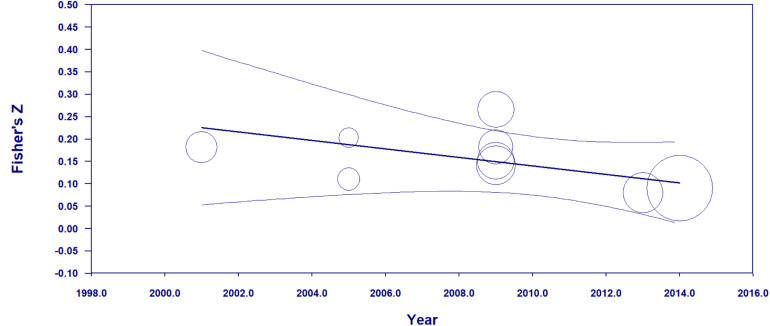
Meta-regression examining the relationship between year of study and the Fisher’s *z* value of the “erasing race” effect size along with 95% confidence internals. Circle size corresponds to the weighting given to each study in the meta-regression. The temporal trend is *b* = –0.010 (*K* = 9, 95% CI = –0.023 to 0.004) indicating no statistically significant change in effect size as a function of year of publication.

A random effects meta-regression of the “erasing race” effects against time was also conducted to investigate the presence of the *decline effect*. This effect stems from the tendency for initial effect sizes to be larger than subsequent ones, which might suggest selective dissemination of more “generous” magnitude effect sizes early in the research paradigm. Alternatively, a change in effect size over time could be an indication of a secular trend. The results of the meta-regression are graphed in Figure 3.

The results of the random effects meta-regression yielded no indications of a decline effect or any other temporal trend (*b* = −0.010, *K* = 9, 95% CI = −0.023 to 0.004).

### Potential for Bias in the Included Effects Due to Recent Methodological Developments

Recent research (Bor, 2018; Pietraszewski, 2018) has revealed a methodological improvement in the calculation of the error base rates behind the effects that were included in the current study. This work correctly identified the potential for bias under the previous method and provided clear and extensive illustrations regarding the mechanics behind an improved method that addresses this problem.

Too few “erasing race” studies have been conducted using the new methodology to form the basis of a distinct meta-analysis; however, it should be possible to synthetically correct the present results for any bias if the magnitude of that bias is known. To determine this, we reanalyzed data from the Pietraszewski (2018) supplement, which recalculated 55 distinct effects (all scaled as *r* values) reported in four of his previous studies. Using these data, we calculated an average across all effect sizes and all studies of the difference between the *r* values computed using the old and the new method. The result is a (effect size number weighted) Δ*r* of −0.07, meaning that the new method tends to produce negatively biased (smaller magnitude) effects relative to the old method. To simulate the likely impact of this to the effect size calculated in our meta-analysis (which was based only on studies employing the pre-2018 methodology), we can use the Δ*r* value from our reanalysis of the Pietraszewski (2018) data to synthetically correct the meta-analytic result (such corrections for error and bias are standard in psychometric meta-analyses; Schmidt and Hunter, 2015). This can help to determine the robustness of the result. Therefore, ρ = 0.137 becomes ρ*_*adjusted*_* = 0.067 when synthetically corrected for the bias between the two methods. This synthetic effect size, if accurate, would still be statistically significant given an *N* of 1432 (95% CI = 0.016 to 0.118).

## Discussion

### Overview

This is the first attempt to meta-analyze the “erasing race” effect, which results from the ability for participants to reduce the degree to which subjects’ “race” forms the basis of participants’ coalition once participants are presented with more accurate information concerning subjects’ actual coalition, such as shirt color corresponding to team membership, etc.

After a meta-analytic search of the relevant literature and correspondence with key researchers in this area, five studies of the “erasing race” effect were identified, containing nine independent effect sizes that could be meta-analyzed. The meta-analysis detected a small-magnitude but statistically significant effect, estimated using a random-effects model. The effects were highly homogeneous also (as they were of very similar magnitude). Eight moderators were identified as being potentially important predictors of heterogeneity across effect sizes; however, given the homogeneity of the effect sizes, the analysis unsurprisingly found no evidence of moderation.

The minimal between-study heterogeneity and lack of evidence of moderator effects might, in part, be a function of the somewhat small number of studies so far conducted and available for meta-analysis on this topic. Although the technical “minimum” number of effect sizes required for a meta-analysis is two, a larger number naturally allows for greater variety among the effects and, therefore, greater meta-analytic power (Weare and Nind, 2011). This limitation is highlighted in our own meta-analysis because in some cases, only single studies presented a contrasting condition (such as in the case of unpublished and preregistered effect sizes or participant sex), reducing the amount of power available for the analysis of the associated moderation patterns.

The funnel asymmetry plot indicated a broadly symmetrical distribution of effect sizes around the midline, which the Egger’s regression confirmed. Duval and Tweedie’s trim-and-fill test, however, indicated that, after adjustment, four “missing” effects were identified to the left of the mean, bringing the overall value of the ρ down to 0.106 (95% CI = 0.061 to 0.151), which is still statistically significant, indicating relatively small potential publication bias. In a study of the “erasing race” effect using socioeconomic status as an alternative coalition across seven Brazilian states, Cosmides et al. (2012) found that the degree to which “race” was suppressed was strongly correlated (*r* = 0.97, *N* = 7 states, 95% CI = 0.805 to 0.995) with the degree to which “race” in the state predicted the social class of the participant. Insofar as a subset of these effects might have yielded null results (in instances in which participant SES was not associated with “race”) this might help to explain the results of our trim-and-fill test, which yielded indications of “missing” effect sizes to the left of the mean. Had these unpublished effect sizes been made available, they might have better balanced our meta-analysis.

The random-effects meta-regression of study effect size against publication year presented no indications of temporal trends across the studies (*b* = −0.010, *K* = 9, 95% CI = −0.023 to 0.004). As noted in the section “Results,” this finding suggests the absence of the decline effect and other temporal trends. But, again, a major limitation here is the relative paucity of effect sizes.

Finally, synthetically correcting the meta-analytic effect size for an aggregate estimate of the negative bias associated with the new Bor (2018) methodology yields a smaller but still potentially statistically significant synthetic effect size (ρ*_*adjusted*_* = 0.067, 95% CI = 0.016 to 0.118). As the pool of “erasing race” studies employing the Bor (2018) methodology increases, a new meta-analysis can be conducted to determine whether the overall result is in line with our synthetic estimate above.

### Theoretical Considerations

An alternative model to the evolutionary-psychological one proposed by Kurzban et al. (2001) and discussed in the Introduction is the Brunswikian evolutionary-developmental theory (Figueredo et al., 2006). This theory posits that there are domain-independent and domain-dependent processes, which regulate the ways in which behaviors are characterized by “independent levels of biological preparedness and plasticity” (p. 211). Based on this model, humans would be expected to exhibit different levels of biological preparedness when dealing with different prospective coalitionary cues. For example, the use of sex as a basis for forming coalitions should be associated with high-levels of biological preparedness and domain-dependent reasoning as the fitness costs to selecting the wrong sex for the purposes of forming social and sexual partnerships are likely to have been very high. If this fitness cost has been relatively invariant across selective history (i.e., the variance in cost is low), then humans should also exhibit relatively little capacity for plasticity when it comes to the ability to learn to use alternative cues (relative to sex) to coalition with artificially heightened salience. The capacity to sample cues for which humans have no biological preparedness *in the absence of ones for which humans are strongly prepared* would present them with a domain-independent problem likely associated with very high variance in fitness cost over time. To deal with this unpredictability, domain-independent mechanisms associated with abstract reasoning would come to play an enhanced role in identifying the differential relevance of such cues to coalition. The ability to detect alliances on the basis of arbitrary social badges of in-group identity (such as fashion and other affectations) should, therefore, be highly ontogenetically plastic with individuals who undergo certain developmental experiences being able to accept a wide range of inputs associated with the sampling of these cues.

In light of this Brunswikian evolutionary-developmental theory, how might humans treat cues related to “race” and ethnicity? An implication of Salter and Harpending’s (2013) critique of Kurzban et al. (2001) is that humans ought to be moderately prepared to use “racial” (or ethnic) alliance cues insofar as these correspond to biogeographic ancestry given that (a) self-identified “race” and ethnicity would seem to be meaningfully related to actual patterns of clustering among morphological and genomic biogeographic ancestry markers, allowing inclusive-fitness benefits from “racially” or ethnically nepotistic behavior; and (b) contra-Kurzban et al. infrequent contact between separate ethnicities throughout the evolutionary histories of these lineages would have presented selective challenges in the EEA and to an even greater extent in the subsequent and far more adaptively significant Holocene epoch, wherein human populations expanded their ranges and would have consequently had far more intense and frequent contact (see Cochran and Harpending, 2009). Being somewhat biologically prepared to utilize cues to ethnic and “racial” affiliation as proxies for biogeographic ancestry in the selection of social and sexual partners might, therefore, have served to increase fitness, especially under conditions of intergroup competition in instances in which those cues serve as hard-to-fake indicators of genetic similarity.

Moderate preparedness does suggest that humans should exhibit some capacity to employ domain-independent forms of cognition to identify more salient alliance cues when biogeographic ancestral group affiliation is less significant to fitness (such as when either interpopulation competition or contact is low). Thus, some behavioral plasticity in terms of cue selection should be present under experimental conditions, in which the degree to which “race” corresponds to coalition can be artificially manipulated, and also under naturalistic settings, in which “racial” and ethnic heterogeneity and/or competition are non-existent (which means that only alternative alliance cues matter).

The idea that there might nevertheless exist some moderate degree of biological preparedness when it comes to encoding biogeographic-ancestry salient coalitionary cues helps to reconcile the findings of this meta-analysis (which are consistent with predictions from Kurzban et al.’s model) with certain other findings that are anomalies for that model. One such anomaly is the observation that infants exhibit a heightened capacity to discriminate between individual faces that correspond to their own “race” compared to those of other “races” (for a meta-analysis of this effect, see Sugden and Marquis, 2017). This finding would be consistent with the idea that humans default to biological preparedness for “race” and ethnicity as proxies for biogeographic ancestry, in the absence of learned information concerning the salience of non-“racial” and ethnic social cues.

The Brunswikian evolutionary-developmental theory also leads to several novel predictions, which would also not be predicted based on Kurzban et al.’s model. For example, the “erasing race” effect may be stronger among so-called WEIRD (White, Educated, Industrialized, Rich, and Democratic) populations (Henrich et al., 2010), wherein levels of individualism are generally higher than the global mean (Fincher et al., 2008) as are the levels of factors of domain-independent conative and cognitive ability. An example of the former might include the General Factor of Personality (GFP), this being the most fundamental dimension of personality, which broadly corresponds to “social efficacy” or the ability to engage in prosocial impression management and socio-monitoring (Musek, 2017) as part of a broader “slow life history” strategy (Figueredo et al., 2011). An example of the latter is general cognitive ability (GCA), which in part captures the ability to solve abstract problems (Jensen, 1998); in evolutionary terms, these can be conceptualized as occurring irregularly across human phylogeny and, thus, constitute evolutionarily novel fitness problems (Geary, 2005). Measured aggregate levels of both the GFP and GCA appear to be higher among Western and also East Asian populations (Eppig et al., 2010; Dunkel et al., 2014), and although there are likely to be a variety of factors that contribute non-trivially to these differences (such as cross-cultural differences in historical parasite load evoking different levels of these traits; Thornhill and Fincher, 2014), one (complementary) possibility is that, in combining higher levels of GFP, GCA, and individualism, Western populations in particular might be simultaneously less biologically prepared and more plastic when it comes to the use of non-“racial” and ethnic cues to alliance than non-Western populations, for which the mean levels of one or more of these traits are potentially lower and background levels of ethno-linguistic fractionalization (as a proxy for intensity of inter-ethnic contact) are typically higher also (Loh and Harmon, 2005). The one study whose findings would have allowed us to examine this dimension of moderation directly (Cosmides et al., 2012) could not be incorporated into the current meta-analysis, however, so this possibility is merely a hypothesis.

Finally, if domain-independent conative and cognitive systems, as reflected in individual differences in the levels of GCA and the GFP, play an important regulatory role in behavioral plasticity as pertaining to alliance cue selection, then the existence of individual differences in the ability to “erase race” might also exist. The observation that negative ethnocentrism (i.e., antagonistic “racialized” social schemata) is more prevalent among individuals exhibiting “faster” life history strategies (which correlate with lower GFP) (Figueredo et al., 2011), and also lower GCA (Dhont and Hodson, 2014), is consistent with this possibility. One of the studies (Moya et al., 2005), which was incorporated into the current analysis, is in fact the only study of the “erasing race” effect to examine individual differences in “race encoding” specifically in relation to both prejudicial and antiprejudicial attitudes. They found no effects on “race” encoding for social dominance orientation (SDO) or for explicit attitudes toward Blacks (ATB); however, lower SDO and positive ATB predicted greater coalition encoding among the males. It was found that “race” encoding decreased with higher levels of self-reported external motivation to control prejudice against Blacks; also greater negative bias toward African-Americans as measured using evaluative implicit association testing predicted reduced “race” encoding, when coalition was not salient.

Experiments in evolutionary psychology are typically designed to examine so-called human universals and, therefore, tend to sample opportunistically (e.g., from student populations) with small- to modestly sized samples. Such sampling is suboptimal for individual-differences research in that, with respect to important dimensions such as GCA, students are range restricted (Russo, 2003). Nevertheless, future research into the “erasing race” effect might follow Moya et al. (2005) in incorporating insights from individual-differences research and may opt to combine this with sampling from the broader population, as Voorspoels et al. (2014) did in their preregistered study to better test these predictions.

## Data Availability Statement

The raw data supporting the conclusions of this article will be made available by the authors, without undue reservation, to any qualified researcher.

## Author Contributions

MW devised the study and wrote the manuscript. MH conducted the meta-analytic search and analysis. MP-A and RB helped to conduct the analysis. MS helped with writing and editing the manuscript. HR helped to writing the manuscript. All authors contributed to the article and approved the submitted version.

## Conflict of Interest

The authors declare that the research was conducted in the absence of any commercial or financial relationships that could be construed as a potential conflict of interest.

## Footnotes

1 We use the term “race” in quotation marks so as to highlight the ambiguities that surround the concept it denotes.

## References

[B1] Barkow J. Cosmides L. Tooby J. (eds) (1992). *The Adapted Mind: Evolutionary Psychology And The Generation Of Culture.* New York, NY: Oxford University Press.

[B2] Bor A. (2018). Correcting for base rates in multidimensional “Who Said What?” experiments. *Evol. Hum. Behav.* 39 473–478. 10.1016/j.evolhumbehav.2018.04.003

[B3] Borenstein M. Hedges L. V. Higgins J. P. T. Rothstein H. R. (2015). *Comprehensive Meta-Analysis, Version 3.* Englewood, NJ: Biostat.

[B4] Buss D. (2011). *Evolutionary Psychology: The New Science Of The Mind*, 4th Edn, New York, NY: Pearson.

[B5] Cavalli-Sforza L. L. (2000). *Genes, Peoples, and Languages.* New York, NY: North Point Press.

[B6] Cochran G. Harpending H. (2009). *The 10,000 Year Explosion: How Civilization Accelerated Human Evolution.* New York, NY: Basic Books.

[B7] Cohen J. (1988). *Statistical Power Analysis For The Behavioral Sciences*, 2nd Edn, Hillsdale, NJ: Lawrence Erlbaum.

[B8] Cosmides L. Yamamoto M. E. Pietraszerski D. (2012). “Erasing race in Brazil: racial categorization varies systematically with patterns of social alliance across seven Brazilian states,” in *Proceedings of the Oral Presentation Given at the 24th Annual Meeting of the Human Behavior and Evolution Society*, Albuquerque.

[B9] Dawkins R. (2004). *The Ancestor’s Tale: A Pilgrimage To The Dawn Of Life.* Boston, MA: Houghton Mifflin Harcourt.

[B10] Dhont K. Hodson G. (2014). Does low cognitive ability predict greater prejudice? *Curr. Direct. Psychol. Sci.* 23 454–459. 10.1177/0963721414549750 22222219

[B11] Dunkel C. S. Stolarski M. van der Linden D. Fernandes H. B. F. (2014). A reanalysis of national intelligence and personality: the role of the general factor of personality. *Intelligence* 47 188–193. 10.1016/j.intell.2014.09.012

[B12] Duval S. Tweedie R. (2000). Trim and fill: a simple funnel-plot-based method of testing and adjusting meta-analysis. *Biometrics* 56 455–463. 10.1111/j.0006-341x.2000.00455.x 10877304

[B13] Edwards A. W. F. (2003). Human genetic diversity: Lewontin’s fallacy. *Bioessays* 25 798–801. 10.1002/bies.10315 12879450

[B14] Eppig C. Fincher C. L. Thornhill R. (2010). Parasite prevalence and the worldwide distribution of cognitive ability. *Proc. Roy. Soc. B Biol. Sci.* 277 3801–3808. 10.1098/rspb.2010.0973 20591860 PMC2992705

[B15] Figueredo A. J. Andrzejczak D. J. Jones D. N. Smith-Castro V. Montero E. (2011). Reproductive strategy and ethnic conflict: slow life history as a protective factor against negative ethnocentrism in two contemporary societies. *J. Soc. Evol. Cult. Psychol.* 5 14–31. 10.1037/h0099277

[B16] Figueredo A. J. Hammond K. R. McKierran E. C. (2006). A Brunswikian evolutionary developmental theory of preparedness and plasticity. *Intelligence* 34 211–227. 10.1016/j.intell.2005.03.006

[B17] Fincher C. L. Thornhill R. Murray D. R. Schaller M. (2008). Pathogen prevalence predicts human cross-cultural variability in individualism/collectivism. *Proc. R. Soc. B Biol. Sci.* 275 1279–1285. 10.1098/rspb.2008.0094 18302996 PMC2602680

[B18] Fiske S. T. Neuberg S. L. (1990). A continuum of impression-formation, from category based to individuating processes: influences of information and motivation on attention and interpretation. *Adv. Exper. Soc. Psychol.* 23 1–74. 10.1016/s0065-2601(08)60317-2

[B19] Garn S. M. (1961). *Human Races.* Springfield, IL: Thomas Co.

[B20] Geary D. (2005). *Origin of Mind: Evolution of Brain, Cognition, And General Intelligence.* Washington DC: American Psychological Association.

[B21] Guo G. Fu Y. Lee H. Cai T. Mullan Harris K. Li Y. (2014). Genetic bio-ancestry and social construction of racial classification in social surveys in the contemporary United States. *Demography* 51 141–172. 10.1007/s13524-013-0242-0 24019100 PMC3951706

[B22] Hamilton D. Stroessner S. Driscoll D. (1994). “Social cognition and the study of stereotyping,” in *Social Cognition: Impact On Social Psychology*, eds Devine P. Hamilton D. Ostrom T. (San Diego, CA: Academic Press), 291–321.

[B23] Harpending H. Harris N. (2016). “Human kinship as a greenbeard,” in *Darwin’s Bridge: Uniting the Humanities And Sciences*, eds Carroll J. McAdans D. P. Wilson E. O. (New York, NY: Oxford University Press), 55–68.

[B24] Henrich J. Heine S. J. Norenzayan A. (2010). The weirdest people in the world. *Behav. Brain Sci.* 33 61–83. 10.1017/s0140525x0999152x 20550733

[B25] Jensen A. R. (1998). *The g Factor: The Science Of Mental Ability.* Westport, CT: Praeger.

[B26] Johnson D. Cesario J. (2013). “Erasing race through recategorization? Additional cues undermine recategorization effects,” in *Poster Presented At The 25th meeting of the Association for Psychological Science*, Washington, DC.

[B27] Jones B. C. Hahn A. C. Fisher C. I. Wang H. Kandrik M. DeBruine L. M. (2018a). General sexual desire, but not desire for uncommitted sexual relationships, tracks changes in women’s hormonal status. *Psychoneuroendocrinology* 88 153–157. 10.1016/j.psyneuen.2017.12.015 29287282

[B28] Jones B. C. Hahn A. C. Fisher C. I. Wang H. Kandrik M. DeBruine L. M. (2018b). No compelling evidence that preferences for facial masculinity track changes in Women’s hormonal status. *Psychol. Sci.* 29 996–1005. 10.1177/0956797618760197 29708849 PMC6099988

[B29] Keita S. O. Y. (1993). The subspecies concept in zoology and anthropology: a brief historical review and test of a classification scheme. *J. Black Stud.* 23 416–445. 10.1177/002193479302300309

[B30] Kurzban R. Tooby J. Cosmides L. (2001). Can race be erased? Coalitional computation and social categorization. *Proc. Natl. Acad. Sci. U.S.A.* 98 15387–15392. 10.1073/pnas.251541498 11742078 PMC65039

[B31] Lewontin R. C. (1972). “The apportionment of human diversity,” in *Evolutionary Biology*, Vol. 6 eds Dobzhansky T. Hecht M. K. Steere W. C. (New York, NY: Springer), 381–398. 10.1007/978-1-4684-9063-3_14

[B32] Lipsey M. W. Wilson D. B. (2001). *Practical Meta-Analysis.* Thousand Oaks, CA: Sage Publications, Inc.

[B33] Littlefield A. Lieberman L. Reynolds L. T. (1982). Redefining race: the potential demise of a concept in physical anthropology. *Curr. Anthropol.* 23 641–655. 10.1086/202915

[B34] Loh J. Harmon D. (2005). A global index of biocultural diversity. *Ecol. Indic.* 5 231–241. 10.1016/j.ecolind.2005.02.005

[B35] Moher D. Liberati A. Tetzlaff J. Altman D. G. (2009). Preferred reporting items for systematic reviews and meta-analyses: the PRISMA statement. *BMJ* 339:b2535. 10.1136/bmj.b2535 19622551 PMC2714657

[B36] Moya C. Navarrete C. Fessler D. Sidanius J. Amodio D. (2005). “Distinguishing patterns of coalition and race encoding: effects of coalition salience, race priming, sex, and individual differences,” in *Poster Presented at the 17th Annual Meeting Of the Human Behavior and Evolution Society*, Austin.

[B37] Musek J. (2017). *The General Factor Of Personality.* London: Academic Press.

[B38] Pashler H. Wagenmakers E. J. (2012). Editors’ introduction to the special section on replicability in pychological science: a crisis of confidence? *Perspect. Psychol. Sci.* 7 528–530. 10.1177/1745691612465253 26168108

[B39] Pietraszewski D. (2009). *Erasing Race With Cooperation: Evidence That Race Is A Consequence Of Coalitional Inferences.* Doctoral dissertation, UC Santa Barbara, Santa Barbara, CA.

[B40] Pietraszewski D. (2018). A reanalysis of crossed-dimension “Who Said What?” paradigm studies, using a better error base-rate correction. *Evolut. Hum. Behav.* 39 479–489. 10.1016/j.evolhumbehav.2018.04.005

[B41] Pietraszewski D. Cosmides L. Tooby J. (2014). The content of our cooperation, not the color of our skin: an alliance detection system regulates categorization by coalition and race, but not sex. *PLoS One* 9:e88534. 10.1371/journal.pone.0088534 24520394 PMC3919763

[B42] Reich D. (2018). *Who We Are And How We Got Here: Ancient DNA And The New Science Of The Human Past*. Oxford, UK: Oxford University Press.

[B43] Russo R. (2003). *Statistics For The Behavioural Sciences: An Introduction.* London: Psychology Press.

[B44] Salter F. Harpending H. (2013). J.P. Rushton’s theory of ethnic nepotism. *Pers. Individ. Differ.* 55 256–260. 10.1016/j.paid.2012.11.014

[B45] Schmidt F. L. Hunter J. E. (2015). *Methods of Meta-Analysis: Correcting Error And Bias In Research Findings*, 3rd Edn, Thousand Oaks, CA: SAGE Publications.

[B46] Sesardić N. (2010). Race: a social destruction of a biological concept. *Biol. Philos.* 25 143–162. 10.1007/s10539-009-9193-7

[B47] Stangor C. Lynch L. Duan C. Glass B. (1992). Categorization of individuals on the basis of multiple social features. *J. Pers. Soc. Psychol.* 62 207–218. 10.1037/0022-3514.62.2.207

[B48] Sugden N. A. Marquis A. R. (2017). Meta-analytic review of the development of face discrimination in infancy: face race, face gender, infant age, and methodology moderate face discrimination. *Psychol. Bull.* 143 1201–1244. 10.1037/bul0000116 28758764

[B49] Tal O. (2012). The cumulative effect of genetic markers on classification performance: insights from simple models. *J. Theoret. Biol.* 293 206–218. 10.1016/j.jtbi.2011.10.005 22004997

[B50] Tang H. Quertermous T. Rodriguez B. Kardia S. L. R. Zhu X. Brown A. (2005). Genetic structure, self-identified race/ethnicity, and confounding in case-control association studies. *Am. J. Hum. Genet.* 76 268–275. 10.1086/427888 15625622 PMC1196372

[B51] Taylor S. Fiske S. Etcoff N. Ruderman A. (1978). Categorical and contextual bases of person memory and stereotyping. *J. Pers. Soc. Psychol.* 36 778–793. 10.1037/0022-3514.36.7.778

[B52] Thornhill R. Fincher C. (2014). *The Parasite-Stress Theory Of Values And Sociality.* New York, NY: Springer.

[B53] Voorspoels W. Bartlema A. Vanpaemel W. (2014). Can race really be erased? A pre-registered replication study. *Front. Psychol.* 5:1035. 10.3389/fpsyg.2014.01035 25278922 PMC4165323

[B54] Weare K. Nind M. (2011). Mental health promotion and problem prevention in schools: what does the evidence say? *Health Promot. Intern.* 26 29–69. 10.1093/heapro/dar075 22079935

[B55] Zack N. (2018). *Philosophy Of Race: An Introduction.* New York, NY: Palgrave MacMillan.

